# Linker Protein Repair of LAMA2 Dystrophic Neuromuscular Basement Membranes

**DOI:** 10.3389/fnmol.2019.00305

**Published:** 2019-12-13

**Authors:** Peter D. Yurchenco, Karen K. McKee

**Affiliations:** Department of Pathology and Laboratory Medicine, Rutgers Robert Wood Johnson Medical School, Rutgers University, Piscataway, NJ, United States

**Keywords:** gene-therapy, self-assembly, adeno-associated virus, muscle, peripheral nerve, extracellular matrices

## Abstract

An understanding of basement membrane (BM) assembly at a molecular level provides a foundation with which to develop repair strategies for diseases with defects of BM structure. As currently understood, laminins become anchored to cell surfaces through receptor-mediated interactions and polymerize. This provisional matrix binds to proteoglycans, nidogens and type IV collagen to form a mature BM. Identification of BM binding domains and their binding targets has enabled investigators to engineer proteins that link BM components to modify and improve their functions. This approach is illustrated by the development of two linker proteins to repair the LAMA2-deficient muscular dystrophy (LAMA2-MD). Dystrophy-causing mutations of the *LAMA2* gene product (Lmα2) disrupt the BM molecular architecture, destabilizing it. In a mild ambulatory type of the dystrophy, α2LN mutations in laminin-211 prevents polymerization. In the more common and severe non-ambulatory type (MDC1A), an absent Lmα2 subunit is replaced by the naturally occurring Lmα4 subunit that is normally largely confined to the microvasculature. The compensatory laminin, however, is a poor substitute because it neither polymerizes nor binds adequately to the anchoring receptor α-dystroglycan. A chimeric laminin-binding protein called αLNNd enables laminins with defective or absent αLN domains to polymerize while another engineered protein, miniagrin (*mag*), promotes efficient α-dystroglycan receptor-binding in otherwise weakly adhesive laminins. Alone, αLNNd enables Lm211 with a self-assembly defect to polymerize and was used to ameliorate a mouse model of the ambulatory dystrophy. Together, these linker proteins alter Lm411 such that it both polymerizes and binds αDG such that it properly assembles. This combination was used to ameliorate a mouse model of the non-ambulatory dystrophy in which Lm411 replaced Lm211 as seen in the human disease. Collectively, these studies pave the way for the development of somatic gene delivery of repair proteins for treatment of LAMA2-MD. The studies further suggest a more general approach of linker-protein mediated repair in which a variety of existing BM protein domains can be combined together to stabilize BMs in other diseases.

## Introduction

Basement membranes (BMs) of skeletal muscle and peripheral nerve are cell surface-anchored extracellular matrices (ECMs) that contain laminins, nidogens, type IV collagen, and the heparan sulfate proteoglycans agrin and perlecan as key structural elements. In muscle, the sarcolemmal BM (sBM) coats and protects myofibers from contraction-induced damage. In peripheral nerve, the endoneurial Schwann cell (SC) BM enables and supports the myelination of axons. A key component of these BMs is laminin-211 (Lm211), a heterotrimeric glycoprotein consisting of α2, β1 and γ1 subunits. Lm211 is required for proper muscle and SC BM assembly. Mutations that ablate expression of the α2 subunit, or that adversely alter Lm211 polymerization, result in a muscular dystrophy that is accompanied by peripheral nerve deficiencies. This disorder is referred to as LAMA2-MD (LAMA2 gene Muscular Dystrophy) and also as MDC1A (Muscular Dystrophy Congenital type 1A). Less common than X-linked Duchenne dystrophy, this autosomal recessive disorder is nonetheless the most prevalent of the congenital muscular dystrophies. Of note, LAMA2-MD is one of several disorders arising from protein-altering mutations now thought to disrupt linkages extending from the stromal-BM interface through laminin to receptors and cytoskeleton that collectively maintain the stability of the sarcolemmal zone (Kanagawa and Toda, 2006).

The muscle symptoms and signs of LAMA2-MD comprise the major component of what is actually a combined muscle-nerve disorder with the disease ranging from severe to mild, depending upon the type of mutation. Patients with complete absence of the laminin α2 subunit, often resulting from nonsense mutations of the LAMA2 gene, are seen to have hypotonia and extremity weakness at birth such that independent ambulation is never achieved. Children with this presentation display a hypotonic posture with splayed legs, facial and extremity weakness, flexed fingers, and foot contractures (Jimenez-Mallebrera et al., 2005; Bonnemann et al., 2014). Difficulty with respiration is common with death occurring in almost a third of patients in the first decade of life if the condition is left untreated. Patients with mutations causing a slight to modest decrease in laminin α2 expression develop a milder form of the dystrophy in which ambulation is achieved. Seen in no more than 5% of patients, the mutations are generally found to be missense or short in-frame deletion mutations. Many of these mutations localize to the N-terminal LN domain and adjacent supporting LE domains of the α2 subunit (Yurchenco, 2015; Yurchenco et al., 2018). It is worth noting that in mice, laminin α2-deficiency is characterized by a prominent sciatic nerve neuropathy as well as a muscular dystrophy. The peripheral nerve-induced atrophy is superimposed on the dystrophic myofiber loss, regeneration and fibrosis of the muscle, with the corresponding nerve phenotype of weakness and paralysis obscuring phenotypic improvements resulting from muscle-specific repairs in the hindlimbs.

In this article, we have set out to review how an understanding of BM assembly at the domain level of interaction can be used for the engineering of linker protein strategies to repair the BM defects of LAMA2-MD. Further, the outcomes of transgenic repair in mouse models have not only advanced the possibility of a new treatment of the human disease, but support important aspects of the model of BM assembly and provide verification of the use of biochemical and cell biological approaches to predict outcomes *in vivo*. The last conclusion is relevant for the approach of engineering of new linker proteins to repair different BM structural defects such as those found in other muscular dystrophies and in Pierson and Alport syndromes. Thus we suggest that the predictive *in vitro* studies and confirmatory mouse outcomes have broader clinically relevant implications.

## Basement Membrane Assembly

**FIGURE 1:**
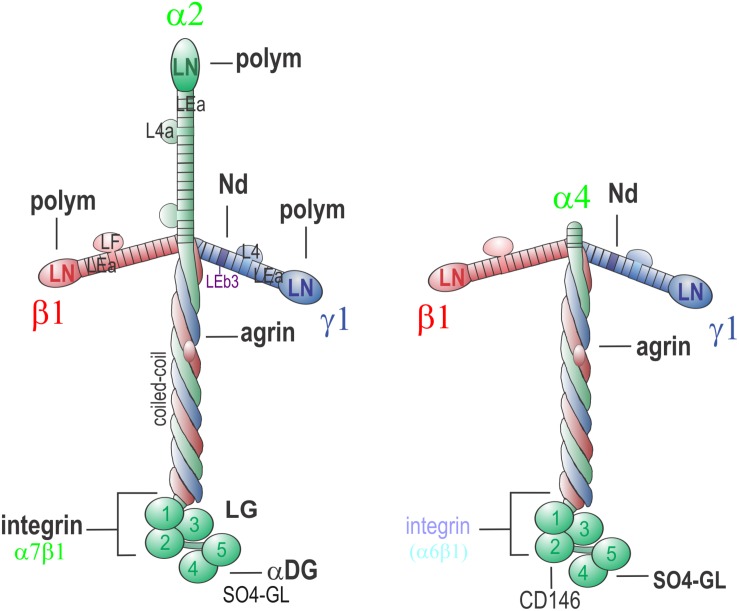
Neuromuscular Laminins. Laminin-211 **(left)** is the principal laminin of the skeletal muscle sarcolemma. Polymerization activity maps to the three LN domains, nidogen-binding to γ1-LEb3, agrin-binding to the γ1 subunit of the coiled-coil, integrin α7β1 to LG1-3 and γ1E1607, α-dystroglycan (αDG) and (in SC BMs) sulfated glycolipids to α1-LG4-5. The LG interactions provide anchorage to the cell membrane and underlying cytoskeleton. Polymerization creates a sheet-like polymer. Nidogen-binding enables strong linkage to collagen-IV while agrin-binding permits additional binding to αDG. In the peripheral nerve BM of the SC sheath that insulates axons, laminin-411 **(right)** co-exists with laminin-211. Lm-411 lacks the short arm of the α2 subunit and hence is unable to polymerize. It also binds weakly to αDG binding and integrins (notably α6β1). In SCs, interactions with CD146 (MCAM) have been described. Laminin-411 is the principal compensatory laminin in muscle in LAMA2-MD.

Laminins are a family of heterotrimeric glycoproteins (Figure 1) that are essential for BM assembly [reviewed in Yurchenco and Patton (2009) and Yurchenco (2011)]. Most laminins have three short arms while a few have only two short arms. This difference is important for understanding the pathogenesis of the common severe form of laminin-deficient muscular dystrophy and how it may be treated.

**FIGURE 2:**
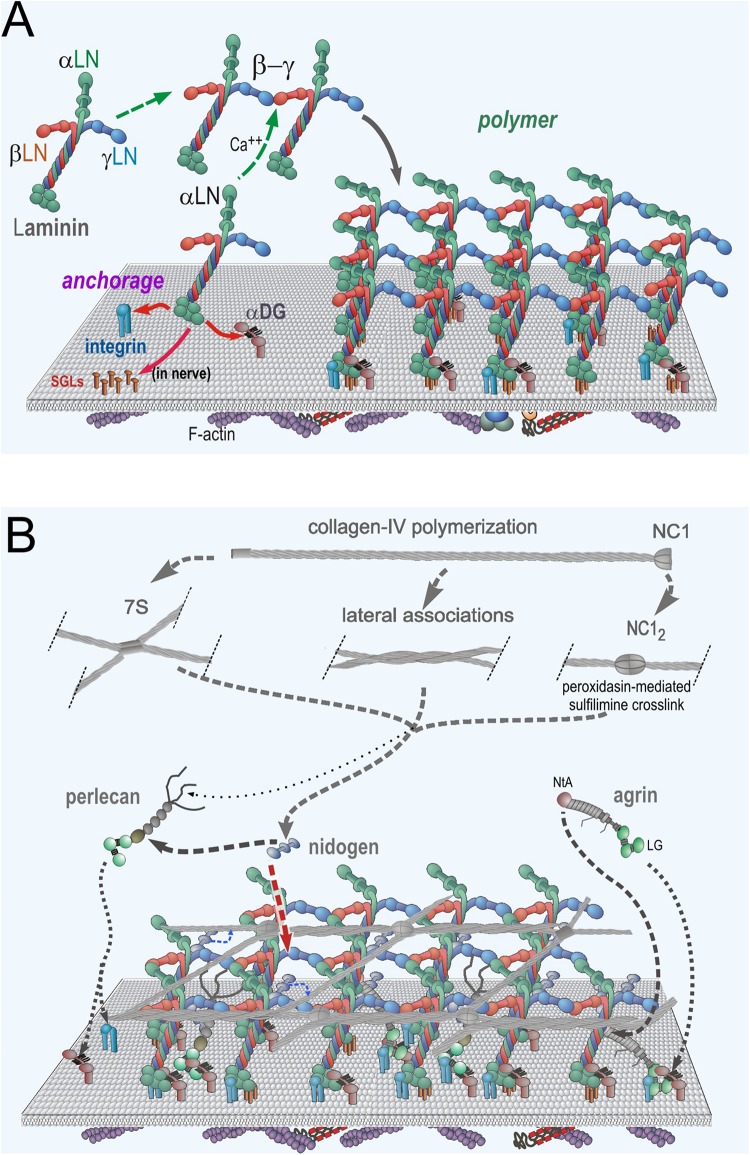
Neuromuscular Basement Membrane Assembly Steps. **(A)** Laminin becomes anchored to the cell surface by binding to integrins (α7β1 in muscle and several laminin-class integrins on Schwann cells) and α-dystroglycan (αDG) receptors. In Schwann cells, there are additional attachments mediated by sulfated glycolipids (SGLs). The three different LN domains of laminins bind to each other to form a planar polymer, creating an initial matrix scaffolding. Lm411 (a normal components of SC BMs) is unable to polymerize. **(B)** The G3 domains of nidogen-1 and -2 bind to the Lmγ1-LEb3 domain. Agrin binds to the coiled-coil domain of laminin and to αDG. Perlecan, another HSPG, binds to the nidogen G2 domain and to αDG. Collagen-IV binds to the G2 nidogen domain and forms a second polymer through covalent N- (7S) and C-(NC1) terminal domain bonds. Non-covalent lateral associations force the collagen into a tighter network.

A body of biochemical, cell and mouse data support a general model of *de novo* BM assembly and resulting structure [reviewed in Yurchenco (2011) and Hohenester and Yurchenco (2013)]. This model (Figure 2) explains how BMs are generated through binding interactions of laminins with cell surface receptors, with themselves, and with other secreted structure-forming components largely through a process of self-assembly. Lm111 (α1-β1-γ1 subunit composition), a well-studied representative of this group similar in domain structure and most interactions to neuromuscular Lm211, is characterized by a series of receptor binding activities that map to the distinct C-terminal moiety. These are the LG1-3 domains that along with the C-terminus of the coiled-coil serves as the key ligand complex for integrin binding (Nishiuchi et al., 2006; Yamada and Sekiguchi, 2015) and the LG4-5 domains in addition to LG1-3 that bind to the mannosyl carbohydrate containing glucuronate-xylose repeats of α-dystroglycan (αDG) (Gee et al., 1993; Hohenester et al., 1999; Smirnov et al., 2002; Briggs et al., 2016). In the case of Lm211 in muscle, it is the α7β1 integrin that provides integrin binding. The receptor αDG has been found to play a more substantial role in mediating BM anchorage to the myofiber as compared to integrin (Han et al., 2009), a difference reflected by the greater severity of the “LARGE” (myd) phenotype compared to the Itga7-mutant genes in mice (Mayer et al., 1997; Holzfeind et al., 2002; Reed et al., 2004; Levedakou et al., 2005).

As laminin molecules attach to receptors of the cell surface, they become concentrated as a two-dimensional layer, favoring polymerization through a process of receptor-facilitated assembly (Colognato and Yurchenco, 2000). This helps create a dense carpet of laminin that enables the binding to, and hence recruitment of, of nidogens-1 and -2, perlecan, agrin and type IV collagen. In this polymerization, the β1 and γ1 LN domains bind to each other followed by addition of the α2 LN domain to form the “polymer node,” a repeating inter-laminin LN complex of the sheet-like laminin network on the cell surface (Yurchenco et al., 1985, 1992; Yurchenco and Cheng, 1993; McKee et al., 2007; Purvis and Hohenester, 2012). Laminins that lack a full complement of LN domains, notably the α4-laminins, are unable to polymerize (Reinhard et al., 2017). This is significant in that Lm411 is the principal compensating laminin expressed in the absence of the Lmα2 subunit (Moll et al., 2001; Reinhard et al., 2017). The nidogens (nidogen-1 is the principal nidogen in muscle and nerve) serve as high-affinity bridges between laminins and type IV collagen and between laminins and perlecan (Fox et al., 1991; Kohfeldt et al., 1998; Hopf et al., 2001). Type IV collagen separately polymerizes into a network through N-terminal (7S), C-terminal (NC1 domain) and weaker lateral interactions (Timpl et al., 1981; Yurchenco and Furthmayr, 1984; Yurchenco and Ruben, 1987; Vanacore et al., 2009; Bhave et al., 2012). Covalent stabilization occurs in the 7S and NC1 domains, while the lateral associations force the collagen polymer into a tight network. Agrin (A0B0 muscle isoform) binds to the γ1 subunit in the laminin coiled-coil domain via its N-terminal NtA domain and to αDG via its C-terminal moiety LG/EGF-like domain complex (Brancaccio et al., 1995; Yamada et al., 1996; Denzer et al., 1998; Kammerer et al., 1999).

### Myofiber and Schwann Cell Basement Membranes

Laminin 211 (α2-β1-γ1) is common to both the muscle and SC BMs (Patton et al., 1997; Holmberg and Durbeej, 2013). However, SC BMs differ from those of the sarcolemma in that (a) Lm411 normally co-exists with Lm211 (Yang et al., 2005), (b) SC adhesion is mediated by several laminin-binding integrins (including α7β1, α6β1, α3β1, αvβ3) compared to only α7β1 in myofibers, (c) sulfatides, capable of adhering to both Lm411 and Lm211 are present on SC surfaces but not detected in muscle (Li et al., 2005), and (d) dystroglycan plays a more central adhesion/anchoring role in muscle (Han et al., 2009), while β1-integrins serve as the principal receptors required for cell polarization and myelination in nerve (Berti et al., 2011).

### Dystrophic Muscle and Peripheral Nerve Basement Membranes

**FIGURE 3:**
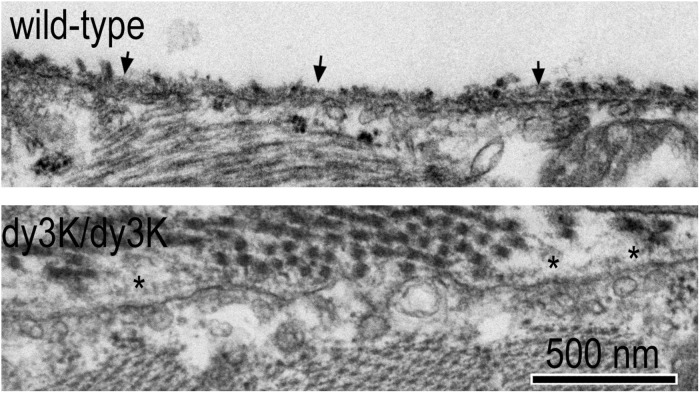
Ultrastructure of normal and laminin-α2-null dystrophic muscle basement membranes. Wild-type and *dy^3K^/dy^3K^* skeletal muscle of mice at 3 weeks of age. Arrows indicate normal sarcolemmal BM with minimal overlying interstitial collagen fibrils. Asterisks indicate attenuated sarcolemmal BM with layer of overlying interstitial collagen fibrils.

The BM ultrastructure of Lama2-deficient dystrophic mouse muscle (Figure 3) reveal focal attenuations (thinning) and denuded regions of the *lamina densa* (electron dense portion of the BM), particularly in the more severe dystrophy of the *dy^*W*^/dy^*W*^* and *dy^3*K*^/dy^3*K*^* mice (Gawlik et al., 2004; McKee et al., 2017; Reinhard et al., 2017). Adjacent to the BM there is an increase in interstitial collagen fibrils, normally sparsely and thinly distributed, that increasingly separate one muscle fiber from the next. An increasing number of myofibers undergo apoptosis and degeneration, inducing chronic inflammation and its sequelae of fibrosis.

With a reduction or absence of the Lmα2 subunit, there is a persistence of expression of laminin subunits that are normally present at only earlier developmental stages. The state of the severely laminin-α2-deficient sarcolemmal BM has been described for *dy/dy*, *dy^*W*^/dy^*W*^*, and *dy^3*K*^/dy^3*K*^* mouse models and is similar among them (Xu et al., 1994; Miyagoe et al., 1997; Reinhard et al., 2017). In the *dy^*W*^/dy^*W*^* and *dy^3*K*^/dy^3*K*^* mice, the laminin α2 epitopes are nearly completely to completely absent while those of the β1 and γ1 are present. In these mice, Lmα4 is substantially increased and laminin-α5 moderately increased (Moll et al., 2001; Reinhard et al., 2017). In the *dy^2*J*^/dy^2*J*^* mice, in which the α2-LN polymerization domain is altered but the remaining protein still expressed, there is a relatively small decrease in the Lmα2 epitope and a modest increase in Lmα4 with minimal changes in Lmα5, accompanied by more modest alterations of BM ultrastructure (McKee et al., 2017). These differences suggest the repair requirements for the mild and severe forms of LAMA2-MD are not identical.

During peripheral nerve development (extending from several days before birth to several weeks of age in mice), SC precursor cells express Lm211 and Lm411, with lesser levels of Lm221 and Lm421, and undergo a laminin- and integrin-dependent process of “radial axonal sorting” in which SC lamellipodial processes extend into, envelop and bundle naked axons followed by their sorting into a 1:1 axon: SC ratio with subsequent myelination (Webster, 1971, 1993). This process is dependent on laminins, collagen-IV, and β1 integrins (Benninger et al., 2007; Nodari et al., 2007; Pereira et al., 2009; Berti et al., 2011). SC BMs also bind to αDG and α6β4 integrins that also contribute to myelination, particularly at later stages of development. Expression of both laminin α-subunits appears to be important for normal myelination of the fibers, suggesting a need for balanced expression may be important (Yang et al., 2005). Absence of the α2 LN subunit (*dy^2*J*^* mouse) was found to result in a prominent radial axonal sorting defect while absence of the α4 subunit was found to result in a less pronounced reduction of sorting with poly-axonal myelination, and absence of both subunits resulted in a particularly severe amyelination. Laminin polymerization, nidogen-binding, and receptor anchorage through β1-integrins were all found to contribute to myelination in organ culture of LamC1-null mouse embryonic dorsal root ganglia (DRG) treated with recombinant laminins (McKee et al., 2012). Of note, the polymerization-dependent deficit of myelination was largely reversed with a linker protein, described ahead, that restores polymerization.

The sciatic nerve and its branches are affected in dystrophic mice to a much greater degree than shorter nerves such as those of the brachial plexus. The myelination phenotype in mice therefore primarily presents as a hindlimb defect of increasing paralysis and extensor contractions that become permanent. The peripheral nerve defect in humans, detected principally as a reduction of conduction velocity, is less severe and sporadic compared to that in mice. An emerging impression is that the peripheral nerve consequences of amyelination are more likely to be recognized in patients with the milder ambulatory form of the dystrophy (Chan et al., 2014).

### Linker Proteins and Repair of Dystrophic Basement Membranes

There are a variety of treatments that are under consideration for amelioration of LAMA2-MD ranging from structural repairs to the inhibition of apoptosis, fibrosis, and inflammation [reviewed in Durbeej (2015), Mohassel et al. (2018), and Yurchenco et al. (2018)]. It seems self-evident that the ideal treatment would be one that completely corrects the structural defect present in the BM early in the disease. One possible approach is to increase the expression of a laminin with properties similar to that found with α2-laminins. It was reported several years ago that transgenic expression of the laminin α1 subunit in *dy^3*K*^/dy^3*K*^* mice substantially rescued both the muscle and peripheral nerve phenotypes (Gawlik et al., 2004, 2006). Furthermore, in a recent study, it was shown that activation of expression of the α1 subunit by CRISPR-dCas9-mediated upregulation of Lama1 improved the dystrophic phenotype of the *dy^2*J*^/dy^2*J*^* mouse (Kemaladewi et al., 2019). This represents a new approach to the treatment of LAMA2-MD.

**FIGURE 4:**
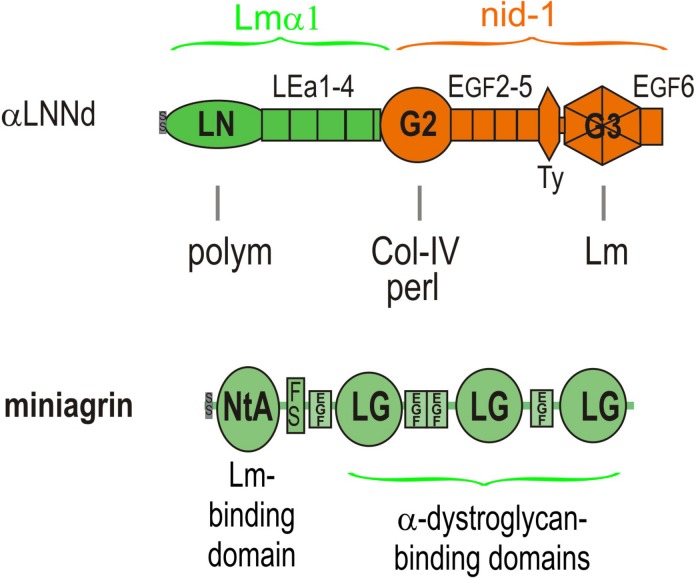
Domain structure of αLNNd and miniagrin. αLNNd is a chimeric protein that consists of laminin α1 LN and adjacent LEa domains fused to the G2 through EGF6 domains of nidogen-1. The G3 domain allows the protein to be attached to a laminin at the nidogen-binding locus located near the origin of the short arms. When attached to a laminin lacking either the native αLN domain or the short arm containing that domain, the αLN domain of αLNNd binds to βLN and γLN domains present on the laminins, enabling laminin polymerization. The G2 domain binds to both collagen-IV and perlecan, activities normally present in nidogen. Miniagrin consists of domains present in non-neural (A0B0) agrin in which the NtA domain and adjacent follistatin domain has been fused to the LG and EGF-like domains of agrin. The NtA domain binds miniagrin to the coiled-coil of laminins while the LG domains bind miniagrin to the α-dystroglycan receptor.

Another approach, the topic of this review, is to modify the defective BM with proteins such that they restore the laminin activities lost in the disease. Two such proteins have been developed and evaluated (Figure 4). One, miniagrin, the first such protein reported (Moll et al., 2001), increases laminin binding to αDG, the key anchoring receptor. The other, αLNNd, enables laminins lacking an αLN domain to polymerize by providing a synthetic short arm carrying a functional αLN domain (McKee et al., 2009). These proteins are sufficiently small in size such that their DNA can be packaged in adeno-associated viruses (AAVs) with appropriate promoters for expression in dystrophic muscle or muscle and nerve. We will discuss αLNNd first and then miniagrin.

**FIGURE 5:**
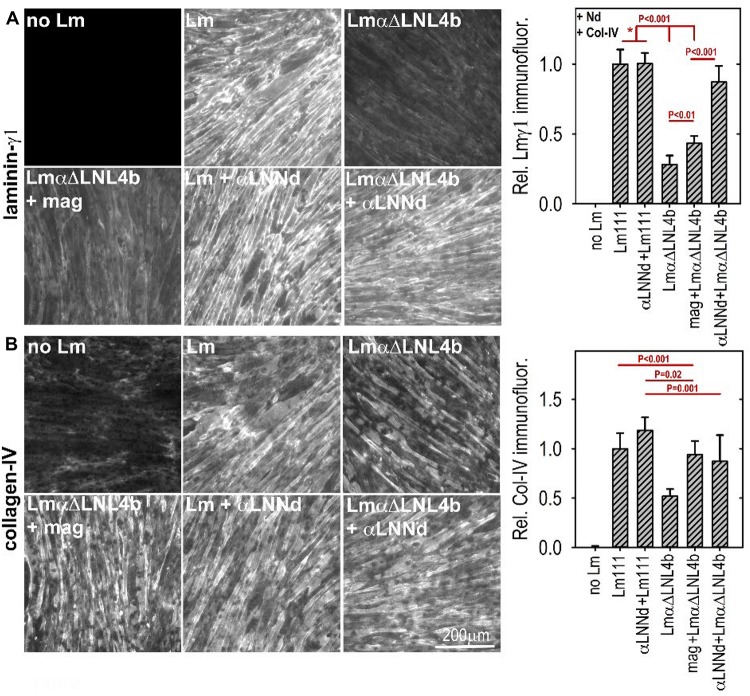
**(A,B)** Laminin assembly on cultured myotubes. Laminin, linker protein and nidogen-1 were incubated at 28 nM separately or together in the presence of 14 nM collagen-IV. αLNNd, bound to the non-polymerizing Lmα1ΔLN-L4b, accumulated on myotubes to a much greater extend compared to that seen in the absence of the linker protein of laminin + miniagrin. Collagen-IV accumulation, which only requires limited amounts of surface-bound laminin, remained high for all linker-laminin complexes (McKee et al., 2017). In a comparison of *dy*^2*J*^/*dy*^2*J*^ laminin without and with αLNNd transgene, it was found that αLNNd increased laminin present in the matrix fraction (McKee et al., 2017).

αLNNd was engineered from the Lmα1 and nidogen-1 subunits and initially used as a tool to dissect laminin polymerization (McKee et al., 2009). It consists of the Lmα1 LN and four adjacent LEa domains fused to the more distal G2 through G3 domains of nidogen-1. The G3 domain binds to the Lmγ1 LEb3 domain near the intersection of the short arms, the LN domain mediates polymerization by attaching to β1 and γ1 LN domains, and the G2 domain enables binding to collagen-IV and also perlecan. The intervening LE (EGF-like domains with 8 rather than 6 cysteines) between LN and G2 and EGF-like domains between G2 and G3 are spacers between the globular domains and hence between bound ligands and, in the case of LEa1-2, enable proper folding and secretion of the LN domain. The expected interactions of αLNNd were confirmed by biochemical characterizations that included direct binding assays, polymerization assays of αLNNd coupled to non-polymerizing versions of recombinant laminin-111, rotary shadow electron microscopy to visualize molecular organization, and cell surface BM assembly experiments on Schwann cells and myotubes (McKee et al., 2009, 2017). The solid phase binding assays revealed that αLNNd bound to immobilized laminin-111 with the same apparent dissociation constant as nidogen-1, that αLNNd bound to immobilized collagen-IV with a dissociation constant slightly greater than that of nidogen-1, and that αLNNd linked laminin to collagen-IV. αLNNd, when coupled to Lmα1ΔLN-L4b, a laminin with only two short arms, appeared as a three short arm laminin in Pt/C rotary shadowed replicas by electron microscopy (McKee et al., 2007). This complex was found to polymerize with a polymerization slope and critical concentration similar to that of wild-type (WT) Lm111, whereas the truncated laminin lacking αLNNd did not polymerize. αLNNd similarly enabled Lmα1ΔLN (which lacks only the αLN domain) to polymerize. The linker-laminin complexes assembled on SC and myotubes (Figure 5) surfaces at levels comparable to wild-type laminin-111 (similar to Lm211), and well-above the levels seen with the non-polymerizing laminins alone (McKee et al., 2009, 2017).

Miniagrin (mag) was derived from the non-neural (A0B0) splice variant of agrin that is normally found in muscle at low levels (Moll et al., 2001). The protein consists of the laminin-binding N-terminal globular domain (NtA) followed by the first follistatin repeat fused to the C-terminal complex of three LG and four EGF-like domains that anchor agrin to the cytoskeleton largely through αDG. The N-terminal NtA globular domain of miniagrin binds to the upper segment of the coiled-coil domain of laminins through a sequence present in the Lmγ1 subunit (Kammerer et al., 1999). The miniagrin-laminin complex was visualized in Pt/C rotary shadowed replica, revealing that miniagrin bound to the coiled-coil domain of the long arm (McKee et al., 2009). LG-αDG binding interactions exist with laminins, agrins and perlecan and are mediated by O-mannosyl carbohydrate chains containing a xylose-glucuronate repeat that are attached to the neck region of αDG (Inamori et al., 2012; Briggs et al., 2016; Hohenester, 2019). This critical interaction creates a link from BM to actin cytoskeleton: αDG is coupled to transmembrane βDG, βDG binds to cytoskeletal dystrophin, and dystrophin binds to the actin-rich cytoskeleton (Matsumura et al., 1993). There are also miniagrin interactions to sulfatides and the α3β1 integrin: however, it is unclear if these interactions are relevant for dystrophy repair (McKee et al., 2007, 2012).

The effect of miniagrin on laminin assembly was evaluated in Schwann cell and myotube tissue culture models of BM assembly (McKee et al., 2009; Reinhard et al., 2017). In SCs, Lm111 lacking all LG domains were unable to adhere to cell surfaces and hence unable to assemble. If this laminin was coupled to miniagrin, the complex accumulated on SC surfaces to a similar degree as WT Lm111 and with a similar concentration dependency. The difference was striking. Miniagrin, expressed as a muscle-specific transgene, was shown to ameliorate the dystrophy of the *dy^*W*^/dy^*W*^* mouse (Moll et al., 2001; Reinhard et al., 2017). Improvements were seen at the histological and ultrastructural level, at the level of laminin expression and resistance to extraction, and at the level of survival, weights, and strength.

In separate studies, the miniagrin gene has been packaged in adeno-associated virus (AAV) and expressed in muscle and nerve and found to ameliorate the dystrophy of the *dy^*W*^/dy^*W*^* mouse (Qiao et al., 2005, 2018). Improvements were seen at the level of tissue histology and in animal performance and survival. The first AAV study revealed selective improvement of muscle. The second study, employing a different AAV serotype and promoter organization, enabled sufficient expression for improvements in both muscle and nerve. Together, these studies demonstrate that AAV can serve as a function-enhancing delivery gene system for a linker protein.

**FIGURE 6:**
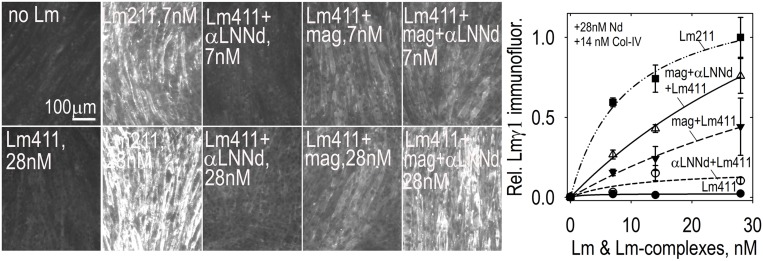
Lm411-linker assembly on myotubes. Lm 411 was incubated without or with bound linker protein(s) at different concentrations in the presence of nidogen-1 (28 nM) and collagen-IV (14 nM). Accumulation on myotubes was assessed by Lmγ1-antibody immunofluorescence. Lm211 produced the highest level of laminin accumulation while Lm411 produced the lowest level. Each linker protein alone elevated Lm411 accumulation. The combined linker elevated accumulation to a level approaching that of Lm211 (Reinhard et al., 2017). An analysis of laminins in the *dy^*W*^/dy^*W*^* mice without and with miniagrin and miniagrin plus αLNNd transgenes revealed that transgene expression improved Lm411 levels and retention within matrix fractions in muscle (Reinhard et al., 2017).

**FIGURE 7:**
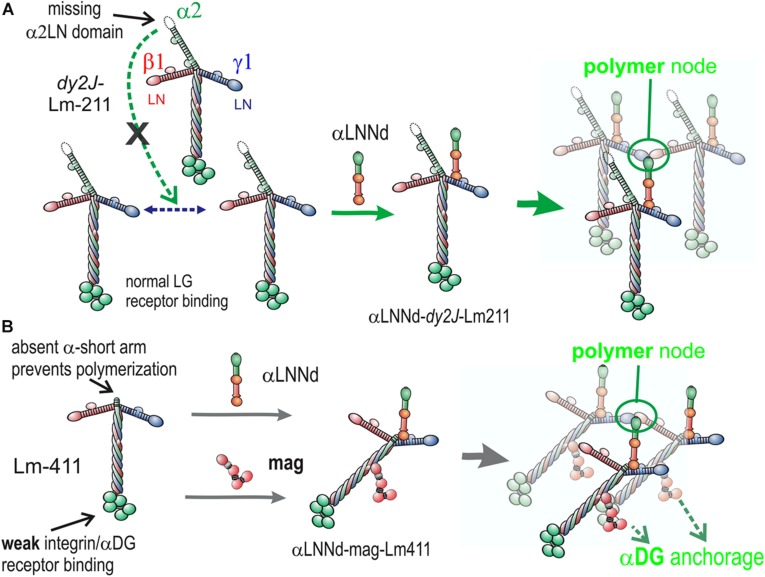
Model of linker protein modification of laminin interactions in models of ambulatory and non-ambulatory laminin α2-deficiency (Yurchenco et al., 2018). **(A)** In the *dy^2J^/dy^2J^* mouse, Lm211 is unable to polymerize as a result of in-frame deletion within the α2LN domain leading to its degradation (Colognato and Yurchenco, 1999). αLNNd binds to the nidogen-binding locus (replacing nidogen), thus “restoring” polymerization. **(B)** In the Lama2 null mice, the compensatory Lm411 neither polymerizes nor binds to the key αDG receptor of muscle. Miniagrin binds to the coiled-coil (replacing native agrin) while αLNNd binds to the nidogen-binding locus. The combination of linker proteins enables polymerization and greatly enhances cytoskeletal anchorage such that the modified laminin behaves in a manner similar to Lm211.

So far we have largely discussed the linker proteins as separate modifiers of BMs. However, single linker alterations of Lm411 in the severe (non-ambulatory) form of LAMA2-MD as a treatment represents an insufficient repair approach given our understanding of the BM requirements of both polymerization and anchorage and the degree of repair observed in mice. If we wish to alter Lm411 so it behaves more similarly to the absent Lm211, further modification is needed. Since both miniagrin and αLNNd proteins and DNA constructs in hand, it seemed logical to use both in a synergistic fashion to affect a change in the activities of the compensatory laminin. SC and myotube BM assembly experiments demonstrated that, at least *in vitro*, the two proteins could bind to the laminin and enable both polymerization and binding to αDG (McKee et al., 2009; Reinhard et al., 2017). The combination of linker proteins was considerably more effective than either alone as promoters of BM assembly on myotube surfaces, approaching levels achieved with Lm211 (Figure 6). The two transgenes were bred with *dy*^*W*^/+mice to generate animal carrying the different alleles for analysis (Reinhard et al., 2017). The two transgenes, each driven by a muscle-specific promoter, were found to improve weights, survival, strength, and muscle histology to a much greater degree than each alone. Importantly, the improvements with the two transgenes effectively phenocopied the benefits observed *in vitro*, ones that could be explained at a mechanistic level in terms of known assembly interactions (Figure 7).

## Future Studies

We have discussed a model of neuromuscular BM assembly that led to hypotheses of how BM defects could be repaired. We traced the path of two engineered laminin-binding linker proteins from biochemical and cell culture analysis to studies conducted in mice following transgenic expression of these proteins. The data resulting from these studies suggest that the human disease the models represent can be ameliorated by similar expression of linker proteins. To do so, the next step is to employ a somatic gene delivery system. AAV is DNA vector delivery system that has shown much promise for the treatment of Duchenne muscular dystrophy (Mendell et al., 2012). The size of the DNAs for αLNNd and miniagrin are sufficiently small to be packaged along with the required viral inverted repeats, promoter and poly(A) tail in the AAV capsid. These viruses, with appropriate choice of serotype and promoter, can be used for combined somatic gene therapy for evaluation of efficacy of repair. It is hoped that such studies will prove fruitful.

Finally, given what is known about BM interactions, it seems reasonable that novel linker proteins can be engineered to repair of a variety of BM disorders in which structure has been compromised. These include other extracellular matrix-based muscular dystrophies, Pierson syndrome (glomerulopathy with ocular disease), and Alport syndrome (glomerulopathy with hearing and later visual losses). While studies are at early stages or even at a speculative stage, several possibilities can be outlined.

Since the discovery of dystrophic mutations in the genes coding for collagen-VI, collagen-IV, laminin-α2, the glycosylation enzymes required for αDG binding, and integrin α7 in addition to those affecting dystrophin, a unifying hypothesis has evolved, i.e., extracellular matrix proteins, their receptors and cytoskeletal partners maintain the sarcolemmal zone through a series of lateral (polymer) and transverse linkages extending from matrix above the BM to the cell cytoskeleton. Mutations adversely affecting these linkages can cause a muscular dystrophy through loss of mechanical stability and dependent signaling (Jimenez-Mallebrera et al., 2005). With this in mind, it may be possible to engineer a variety of proteins to replace lost linkages, e.g., to enhance collagen-VI stability in Bethlem myopathy by crosslinking the collagen to itself or to collagen-IV with a dimerized binding protein. However, one limitation of the linker approach worthy of mention concerns the generation of proteins that bind to the α7β1 or α6β1 integrins to enhance compensatory laminin adhesion. This restriction stems from the finding that integrin binding requires parts of all three laminin subunits, i.e., the distal coiled-coil and LG1-3 (Pulido et al., 2017; Taniguchi et al., 2019).

Other BM diseases can be addressed as well with this approach. In a subset of Pierson syndrome patients, the disease results from laminin β2LN mutations that cause a failure of laminin polymerization (Funk et al., 2017). Polymerization can be restored by βLNNd, a homolog of αLNNd that enables polymerization of laminins with βLN mutations (McKee et al., 2018). In Alport syndrome, there is a reduction of disulfide covalent crosslinked collagen due to loss of α3/α4/α5-collagen-IV (Gunwar et al., 1998). Here it may be possible to stabilize the BM by crosslinking the residual α1/α2 collagen-IV network with a dimerized collagen-binding protein domain such as nidogen G2. Finally, new insights into linker proteins will likely arise as we continue to elucidate how the BM and its stromal and receptor partners interact.

## Author Contributions

PY wrote the initial draft of the manuscript. KM carried out most of the αLNNd studies reported therein.

## Conflict of Interest

The authors declare that the research was conducted in the absence of any commercial or financial relationships that could be construed as a potential conflict of interest.
